# Supplementary figures and images for: Analysis of low-level somatic mosaicism reveals stage and tissue-specific mutational features in human development
Source: PLoS Genet. 2022 Sep 19;18(9):e1010404. doi: 10.1371/journal.pgen.1010404 (PMC9560606; doi:10.1371/journal.pgen.1010404)

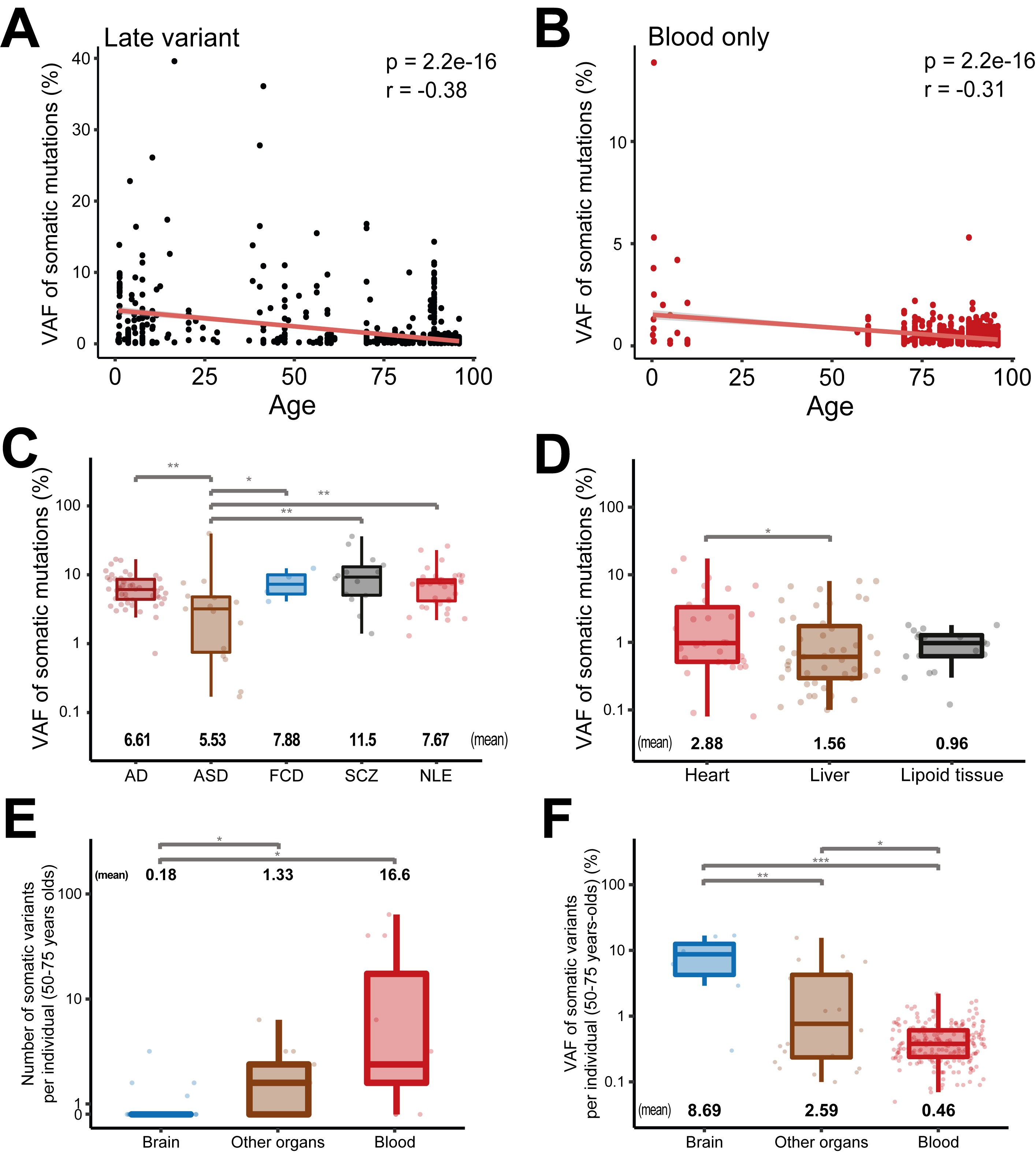

Supplement: S1 Fig — Late mutational analysis by mutagen in different organs (brain, blood, other organs) (A), (B) Age correlation with somatic mutation VAFs in late-stage variants and blood late-stage variants. (C) VAFs of late-stage somatic mutations in brains are classified by the diseases in individuals. (D) VAFs of late-stage somatic mutations in the heart, liver, and lipoid tissues. (E), (F) Number of mutations per individual and VAF distribution in individuals aged 50–75 years. (TIF) [file pgen.1010404.s001.tif]
