# Supplementary material for: Analysis of low-level somatic mosaicism reveals stage and tissue-specific mutational features in human development
Source: PLoS Genet. 2022 Sep 19;18(9):e1010404. doi: 10.1371/journal.pgen.1010404 (PMC9560606; doi:10.1371/journal.pgen.1010404)
Supplement: Attachment — Submitted filename: Response to Reviews_0409_JHK_swkim_0613_final.docx [file pgen.1010404.s004.docx]

**-Response to Reviews-**

**Reviewer #1**

**General Comment:** The authors present an interesting study that leverages whole-exome sequencing (WES) data from multiple tissues from a large number of individuals (190) to gain insight into the nature of somatic mutations that occur in early and late embryonic development. The authors classify somatic mutations as early if they are shared by two different tissues and late if they are not. In addition, the authors also sequenced a subset of brain tumors for comparison. The main finding is that early mutations have higher allele frequency and lower functional impact than late mutations as revealed by multiple genetic metrics (lower dN/dS, location in trinucleotides with atypical mutability, and location in LoF-tolerant genes). In addition, novel mutational signatures and their contributions to different brain regions are described. While the approach is interesting and there is a high need in the field for this sort of comparative analysis, the article has some important caveats, described below.

**SUMMARY of the response to the reviewer 1:**

We thank the reviewers for their thoughtful comments and point-by-point responses to the Reviewer’s comments are given below. Changes to the manuscript have been highlighted in yellow in the revision.

**Q1.** The study is far from being a systemic and comprehensive analysis of low-level somatic mutations, as claimed in the abstract. Only 3 tissue types (brain, blood, and liver) are analyzed in a large number (60+) of individuals. Authors claim multi-organ sequencing but for most individuals, only two tissue types are analyzed: either brain and blood, or brain and liver. The authors should tone down the claim of multi-organ analysis since the study is essentially on brain tissue, just using different control tissues.

**Response to Q1:** We agree with the reviewer that our article focused on three main tissues: brain, blood and liver, while sources from other organs are limited. Therefore we revised our article to resolve this confusion like the below corrections. We thank the reviewer for the comments.

1. We changed the title of our manuscript to “Analysis of low-level somatic mosaicism reveals stage- and tissue-specific mutational features in human development.” (Page 1, line 1-2, revised manuscript), by removing the word “multi-organ”.
2. We revised the below parts of the main text not to emphasize the multi-organs.
   a. Our cohort consisted of two or more organs, including brain and matched-peripheral tissues, and covered a variety of neurologic conditions. Thus, these pairwise analyzes of the diverse individuals’ age, brain locations, and diseases allow for multi-dimensional analysis and particularly direct comparisons with cancer mutations identified from the same analysis procedure. (Page 5, line 87-91, revised manuscript)
   b. In summary, based on a large scale of deep whole exome sequencing data using a total of 498 matched sample pairs in 190 individuals, we provided a more detailed picture of low-level but clone-forming somatic mutations, the counts, and characteristics of which are distinguished by time and space. (Page 11, line 2010-204, revised manuscript)
   c. By pairwise analysis of the brain and other matched organs, we outlined confident mutational counts, variant fraction, and mutational process at each developmental or occurred stage. (Page 11, line 211-213, revised manuscript)

**Q2. (#1)** The article is difficult to read because some key points are not clearly explained. For example, in the 19 individuals with brain tumors, the brain tissue analyzed was from tumor or from normal? That is unclear in figure 1 and it is not clearly explained in Material and Methods. The text only mentions: “If the source of the sample was related to a brain tumor, it was separately regarded as tumor mutations.” Please clarify the origin of samples in individuals with cancer in Fig. 1A. **(#2)** Please clarify whether brain tumor tissue was used for the case-control comparisons explained in Fig. 1A. If it was used, this could be a source of confounding because in individuals with cancer, shared mutations tumor-blood could be due to ctDNA or cancer cells disseminated in the blood stream.

**Response to Q2 - #1:** We agree with the reviewer that there were some key points that were not clearly explained. We thank the reviewer for the comments. We revised the manuscript to resolve the unclear explanations including the main text and Fig. 1. The corrections are listed below.

1. Tumor specimens were collected from the primary or recurred tumor and evaluated microscopic tumor involvement by the histological exam, and microscopic normal brain or blood was used as a control sample. (Page 17, line 338-340, revised manuscript)
2. Tumor specimens were acquired from the primary tumor, and microscopic tumor involvement by the histological exam was evaluated by a neuropathologist according to World Health Organization (WHO) 2016 grading criteria [17]. (Page 6, line 106-109, revised manuscript)
3. Tumor mutations are originated from primary or recurred tumor specimens, confirmed by histologic examination. (Figure 1 legend, revised manuscri

**Response to Q2 - #2:** Yes, brain tissues were also used as control in finding somatic mutations in normal tissues. We first revised the manuscript to more clearly explain the procedure for mutation calling in Figure 1 legend and main text.

1. To find somatic variants, Mutect2 and RePlow/NeuSomatic were used for reciprocal mutation calling by all-pairs testing; first, to detect somatic mutation of tissue “A”, tissue “A” and “B” were used as a case and control, respectively, then tissue “A” was used as a control to identify somatic mutations of tissue “B”. (Page 18, line 368-370, revised manuscript; Figure 1 legend, revised manuscript)

We understand the concern about the potential inclusion tumor somatic mutations in matched normal tissues via ctDNA. It is true that plasma ctDNA can be released into the blood-stream from primary tumor within a patient, especially in patients with advanced or metastatic disease. However, we think that the contamination from ctDNA in our study is unlikely for the following reasons. First, in the aspect of pre-analysis, ctDNA is usually highly fragmented (average length of 167 bp, range 35 to >10000bp) and easily lysed with a half-life 2 hours, from 16 minutes to a few hours [1-3]. A major challenge of liquid biopsy is the detection of low concentration of ctDNA, thus the large volume of plasma is the preferred source for ctDNA detection [2, 4]. In addition, timely ctDNA collection is critical to avoid cell lysis. However, in our study, a conventional blood extraction kit (Wizard genomic DNA purification kit, Promega, Madison, WI, USA) was used without plasma isolation, and we confirmed the size of the extracted gDNA within mostly 100-200 kbp by gel electrophoresis. Secondly, a recent study of genomic alterations in ctDNA in 21,000 advanced cancer patients revealed that the median variant allele frequency for somatic SNVs of ctDNA was 0.4%, and the blood-brain barrier limits ctDNA to enter the peripheral bloodstream in the brain tumor [5]. To identify ctDNA mutations, the critical advantage of targeted NGS compared to whole genome or exome sequencing is its robustly increased coverage depth, thus most studies used deep targeted cancer gene panels of more than 10,000X. A combination of optimized pre-analytic, sequencing, and analytic algorithms are often used to achieve better performance of NGS assays for low-abundance sequence variants in ctDNA. Collectively, it is less likely to find shared mutations in ctDNA with an average of 6.17% of VAF using 500X conventional WES without an optimized ctDNA extraction or analysis method.

**Q3.** The legend for Fig. 1A needs more explanation, especially regarding what samples are considered cases vs controls and the one-sample proportion test. What are the tests for organ specificity?

**Response to Q3**: As the reviewer suggested, we revised the Fig. 1A legend to describe more details about mutation calling process. Briefly, we used the “all pair testing” approach for mutation calling, wherein somatic mutations were called using all possible pairs. For example, in the case of tissue A and B in an individual, two mutation calling processes were done: calling mutations in A by using B as control, and calling mutations in B by using A as control.

We also revised the manuscript to clarify the test for organ specificity.

1. We determined allelic frequency concordance using a one-sample proportion test. One-sample proportion test provides an individual’s difference based on a binomial proportion, which is used for a testing hypothesis about a hypothesized proportion in comparison to a given theoretical proportion of the population. Thus, we tested whether there is a difference between the individual’s allelic frequency of the somatic mutations and that of the population. (Page 19, line 384-389, revised manuscript)
2. After strict filtration and tests for organ specificity by one-sample proportion tests (Fig 1A; Methods), we detected 103 early- and 997 late-stage mutations, as well as 583 tumor mutations. (Page 6, line 112-114, revised manuscript)

**Q4. (#1)** When quantifying the number of early and late-stage somatic mutations per individual, how do the authors correct for the fact that different number of samples and different tissues were analyzed in different individuals? **(#2)** The article might benefit from removing the ‘other tissues’ and ‘heart’ subgroups since there are not many samples in those subgroups and will make the comparisons more similar across individuals. The different sample types used for different individuals is a concern.

**Response to Q4-#1**: As the reviewer commented, our analytic method inevitably increase with more organs. Therefore, we corrected by dividing the number of detected mutations by the number of tissues. Corrections regarding the concerns were made on the manuscript as below:

1. Since the different number of samples analyzed can lead to bias, we corrected by dividing the number of detected mutations by the number of tissues. (Page 19, line 398-399, revised manuscript)
2. On average, there were 0.18 early- and 2.8 late-stage somatic mutations per individual (Fig 2A). Apparently, the numbers of mutations in normal tissues were substantially lower than those of tumors (8.12 per individual). The overall numbers of the late-stage and tumor mutations positively correlated with age (Pearson’s *r:* late-stage, 0.45; and tumor, 0.36) (Fig 2B). (Page 7, line 122-126, revised manuscript)
3. The numbers of mutations varied substantially by organ, with a smaller number in the brain (0.67 per individual) and a higher number in the blood (9.23 per individual) relative to other peripheral organs (average: 1.12 per individual) (Fig 4A). (Page 9, line 164-167, revised manuscript)
4. The number of late-stage mutations and the age of individuals showed a significant positive correlation (*r*=0.45; *p*=2.99×10^−10^) in only the blood (Fig 4C), which has been well-documented by post-natal clonal hematopoiesis [6, 7]. (Page 9, line 166-169, revised manuscript)
5. The numbers and variant fractions of mutations substantially varied by organ, and in the brain, late-stage mutations occurred fewer mutations (0.67 per individual), but high VAF (7.32%). (Page 13, line 248-250, revised manuscript)

**Response to Q4-#2**: Although we are different organs, we performed an analysis including "other tissues" because we thought that they shared common characteristics and that they were related to aging and general intrinsic processes. In other words, by adding “other organs” as well as the brain and blood in this way, the characteristic signature of each organ would be weakened and the proportion of common features could be preserved. Thus, it was better to use a mixture of different organs to find common mutagenic processes of the overall body. Current studies using comparing tissue samples across organs from the same individual have shown that mutation burdens and mutational signatures and degrees of clinical expansion would be varied [8]. However, endogenous mutational processes with SBS1 and SBS5 mutational signatures are ubiquitous among normal tissues [8] as well as cancer.

**Q5. (#1)** It is unclear how the mutational signature analysis might be relevant when the late-stage mutations come from a mixture of different tissues including brain, heart, liver, blood and others. Different tissues harbor different mutational tissues, please see PMID: 34433965 and 34433962. (#**2)** In addition, the number of early-stage mutations is small (94) for robust mutational signature analysis.

**Response to Q5_#1:** Current studies using comparing tissue samples across organs from the same individual have shown that mutation burdens and mutational signatures and degrees of clinical expansion would be varied [8]. Somatic mutations in normal tissues are associated with aging and disease. However, endogenous mutational processes with SBS1 and SBS5 mutational signatures are ubiquitous among normal tissues [8] as well as cancer [9], but exogenous mutational processes operate highly variable in multiple tissues even in the same donor [8].

In somatic mutation studies of brain tissues, somatic mutations have been accumulated by age-, region-, and disease-related manners [10], and C>T and T>C variants were most dominantly found, especially T>C variants increased with age, linked to oxidative stress [10, 11]. Also, the spectrum of mutations in hematopoietic stem cells was dominated by C>T and T>C transitions [12]. In normal liver, ubiquitous mutational signature (SBS5) were prevalent and accumulated linearly with age. Normal liver shared a signature dominant to blood stem cells, which is presumed to be caused by an endogenous mutational process [13]. In cardiomyocytes as well, the contribution of C>T and T>C mutations increased with age [14]. Collectively, although we included different tissue types, many studies show that predominant mutational processes occur and accumulate endogenously, and these mixed signatures may rather reduce mutational bias by each organ.

**Response to Q5_#2:** Compared to late mutations, early mutations occur at a lower rate. So, we described our study limitations and referred to other previous studies of mutational signatures in the early stage.

1. Nevertheless, due to the small total number of early mutations, the signature analysis and its interpretation require caution, but early shared mutations in previous work showed comparable results with our data [11]. (Page 12, line 244-Page 13, line 246, revised manuscript)

**Q6.** The comparison of number and VAF of late-stage somatic mutations by tissue type should take into consideration the fact that the 3 groups of tissues are not age-matched. There is no data for older individuals for ‘other organs’.

**Response to Q6:** We thank the reviewer for the valuable comments. As the reviewer suggested, older individuals in ‘other organs’ do not exist due to sampling limitations. Despite such limitations, when comparing only patients aged 50 to 75 years who can age-match, there is a slight change in the value, but the trend is maintained (Below figures). Therefore, our data could represent the overall late-stage somatic mutations.

**Q7.** The brain tumors included in the study are glioblastoma and ganglioglioma. Glioblastoma is an adult tumor and ganglioglioma is a child tumor. Is it possible that the unique tumor mutational signature identified is the result of the mixture of mutations derived from these two very different brain tumors? If the ganglioglioma tumors were removed, would the mutations observed resemble to the landscape of mutations (driver genes) and mutational signatures reported for glioblastoma?

**Response to Q7:** The mutations of GBM occupy a large number of tumor mutations due to its nature of high mutational load and adult-aging development compared to GG. However, since the number is small, it is difficult to separate and analyze it by itself. When comparing the tumor mutational patterns and cosmic signatures with the exception of the GG samples, there was no significant differences. Thus, we thought that tumor signature corresponds to the GBM signature (Below figures).

Q8. Very low resolution of figures makes it impossible to read some of the content.

**Response to Q8:** We revised high resolution of figures.

**Reviewer #2**

Hyeonju Son and associates analyzed mosacism for SNVs and indels by means of deep whole-exome sequencing in 498 tissues from 190 individuals. Their findings were consistent with those of previous studies: somatic mutations with apparent early embryonic origin were fewer in number but had higher variant allele frequencies than variants that appear to have arisen later in life. Mutational signatures among early embryonic and later-arising variants were similar but differed somewhat.

This research is well described, and the paper is generally well written, although there are occassional errors of English usage. There are a few issues that require attention:

**SUMMARY of the response to the reviewer 2:**

We greatly appreciate the reviewer’s questions and comments on the manuscript. We found that the comments of the reviewer are extremely important and were seriously considered in the preparation of this revision. We would you like to note that we have summarized the things pointed out as point-by-point answer form.

**POINT by POINT answers to the reviewer 2:**

**Q1.** Line 233: "Early-stage mutations were not functionally tolerated than those of late-stage mutations." What does this mean? Are some words left out?

**Response to Q1:** Thank you for your comment, we revised that for a better understanding.

1. Early stage mutations were less functionally tolerated than late stage mutations. Mutations arising early in development can be propagated to a large fraction of the body. In other words, since the early stages are critical to development and can even be embryonic-lethal, it can be inferred that mutations occur in less functionally essential regions. (page 12, line 237-240, revised manuscript)

**Q2.** Discussion: The authors should discuss the limitations of this study (limited range of tissues studied, limited numbers of tissues per patient, inability to detect some kinds of variants such as CNVs, etc.).

**Response to Q2:** As the reviewer commented, we revised main text.

1. We have several limitations. Our study performed using limited the range and numbers of tissues per individual, and the total number of mutations was small due to the application of exome-level sequencing. Thus, it was difficult to find sufficient SNPs or CNVs to analyze. We analyzed tissues from different donors with distinct germline or disease-susceptible backgrounds and lifestyles, thus making a mutagenesis interpretation challenging. To address these issues, current studies have been performed on sequencing in multiple tissues from the same donor [8]. Therefore, as in our study, research conducted through the mixture of multiple organs and different individuals is likely to weaken the characteristics of individuals’ mutation processes and highlight the results of common mutagenesis. Therefore, although the common characteristics of each tissue were analyzed, it is true that the analysis of external and internal mutagenesis is difficult. (page 13, line 268-page 14, line 278, revised manuscript)

**Q3**. Line 292: "Subjects with refractory epilepsy, including focal cortical dysplasia and non-lesional epilepsy, and who had undergone epilepsy surgery were enrolled through the Severance Children’s Hospital of Yonsei University College of Medicine." If these samples were obtained because of regional brain tissue excision for intractable epilepsy, could the mutations found have been responsible for the seizures? If so, might the frequency of variants and/or the VAF be biased to higher values in these samples? The same question could be raised in any other group of samples obtained by surgery to remove pathological tissue. This is an issue that the authors should discuss.

**Response to Q3:** We thank the reviewer for the valuable comments. As your concern, our study included samples obtaining regional brain tissue excision for intractable epilepsy, and this numbers of variants and/or the VAF be biased. Thus, we measured VAF of FCD and NLE group compared to remaining disease group. However, there were no significant difference: average VAF 7.1% in FCD/NLE and 6.5% in others (*p*-value=0.220, Mann-Whitney U test). We also checked the other disease including Alzheimer’s disease (6.5%) and schizophrenia (VAF= 8.3%) or other disease (VAF = 6.7%), but there was no significant difference in VAF value. (p-value=0.14 and 0.92 each, Mann-Whitney U test). Therefore, it appears that sampling from the lesions did not significantly affect VAF.

**Reviewer #3**

Throughout its lifetime, human body accumulates mutations, which despite affecting multiple body parts rarely cause a pathology. To provide a better grasp of spatiotemporal characteristics of this genetic mosaicism, the Authors of the manuscript performed deep whole exome sequencing of human tissues comparing the pattern of clone-forming mutations in various organs. In its focus on differences between early (pre-gastrulation) and late (post-gastrulation) mutations, this well-designed study addresses an interesting, and largely unexplored problem of developmental shifts in somatic mutagenesis and makes up an important contribution to current studies about somatic mosaicism.

**SUMMARY of the response to the reviewer 3:**

We are sincerely grateful for reviewer’s comments and positive evaluation of our manuscript. The review’s comments helped us to have more objective point of view, to have extended perspectives for some matters we have been considering during our study, and to make it a more complete manuscript. Our detailed point-by-point answers are provided below.

**POINT by POINT answers to the reviewer 3:**

1. **Reviewer’s comment:**

Q1. The results include the finding that most somatic mutations are produced after gastrulation and that diversity of late-appearing mutations is higher than of the early ones. However, it is not clear if the observed numerical differences between early and late clone forming mutations reflect distinct mutation rates during these developmental stages. More details should be also provided to elucidate whether differences in mutational signatures across tissues stem from the action of distinct (internal and external) mutagens in these sites. They Authors should refer to recent studies by Li et al., Nature 2021, 597 (7876): 398-403, who also considered the problem of genetic mosaicism and compared the mutational pattern across tissues.

**Response:** Thank the reviewer’s good comments. As you commented, we obtained more numbers of mutations in the late-stage than those of early-stage’s. As recent studies by Li et al., Nature 2021, 597 (7876): 398-403, they deciphered seven mutational signatures, including two age-related endogenous mutational signatures, SBS1 and SBS5 [8]. The relative activities of SBS1 and SBS5 varied across tissues, in particularly duodenum, colon and rectum showed higher SBS1/SBS5 ratio than the bronchus, pancreas, esophagus, and liver [8]. In addition, they described that exogenous mutational signatures exhibited organ- and individual- dependency [8]. As the reviewers pointed out in our study, nevertheless, it is difficult to conclude that such a difference in numbers represents the mutation rate in the early and late developmental stages. The reason is (i) it is difficult to determine the exact amount due to the difference in detectability (e.g., VAF) of clone forming mutations occurring in early and late periods. (ii) The number of early and late mutations measured by our method is affected by the number and location of matched tissue within an individual, so it is difficult to conclude that these numbers are representative. (iii) It is difficult to convert to an accurate mutation rate because the time difference between each stage and the rate of cell replication/differentiation are different from the time when we distinguish between early and late. Therefore, as the reviewer pointed out, we need to be careful in concluding that the mutation rate is high in late and they are dominant, and the above is reflected in discussion section of the revised paper.

1. As recent studies by Li et al., Nature 2021, 597 (7876): 398-403, they deciphered seven mutational signatures, including two age-related endogenous mutational signatures, SBS1 and SBS5 [8]. The relative activities of SBS1 and SBS5 varied across tissues, in particularly duodenum, colon and rectum showed higher SBS1/SBS5 ratio than the bronchus, pancreas, esophagus, and liver [8]. In addition, the described that exogenous mutational signatures exhibited organ- and individual- dependency [8]. We obtained more numbers of mutations in the late-stage than those of early-stage’s. Nevertheless, it is difficult to conclude that such a difference in numbers represents the mutation rate in the early and late developmental stages. The reason is (i) it is difficult to determine the exact amount due to the difference in detectability (e.g., VAF) of clone forming mutations occurring in early and late periods. (ii) The number of early and late mutations measured by our method is affected by the number and location of matched tissue within an individual, so it is difficult to conclude that these numbers are representative. (iii) It is difficult to convert to an accurate mutation rate because the time difference between each stage and the rate of cell replication/differentiation are different from the time when we distinguish early and late. Therefore, we need to be careful in concluding that the mutation rate is high in late and they are dominant. (page 14, line 279-page15, line294, revised manuscript)

Q2. **:** I see the value of the present manuscript in its potential to contribute to ongoing studies of clonal evolution and suggest that the Authors elaborate on how their work contributes to recent studies on this subject (Kakiuchi and Ogawa, Nat Rev Cancer 2021, 21(4):239-256; Swiatczak, Cell Mol Life Sci, 2021 78(21-22):6797-6806; Laconi et al. 2020, Br J Cancer 2020, 122(7):943-952). For example, the Authors hint that variant allele frequency (VAF) can be used as a measure of a degree of clonal expansion of mutated cells in various tissues but draw no conclusions pertaining to the question of somatic evolution based on the observed differences between VAF in the brain, blood and other tissues.

Response to Q2: We thank the reviewer for the valuable comments. In our study, the early- and late-stage mutation signatures may have been influenced by the tissue used as well as the characteristic signature for each period. Our body continues to be remodeled by positively selected clones according to the survival of the fittest principle under given ecosystems, which is thought to set a stage for cancer evolution as well as even in normal ageing and the development of non-neoplastic diseases [15, 16]. The degree of remodeling substantially differs depending on the tissue type and the ecosystem, and external mutagen such as chronic inflammation, alcohol consumption and tobacco smoking can affect on it [15]. In addition, microenvironmental decline, progressive decline of competitive fitness of the tissue, and relatively low levels of clearing altered (dysfunctional) cells have been seen in with aging tissues, some of which can be oncogenic [17]. In the case of the brain during somatic evolution, cellular division/differentiation is relatively slow or almost quiescent, thus mutations that occur early in development are less frequent, but remain high in VAF. On the other hand, in the case of blood and other tissues, continuous differentiation proceeds, and if positive selection does not occur due to a special driver mutation, it can be speculated that it will have a relatively low VAF with a high number of mutations while having similar cellular fitness. the above is reflected in discussion section of the revised paper.

1. Our body continues to be remodeled by positively selected clones according to the survival of the fittest principle under given ecosystems, which is thought to set a stage for cancer evolution as well as even in normal ageing and the development of non-neoplastic diseases [15, 16]. The degree of remodeling substantially differs depending on the tissue type and the ecosystem, and external mutagen such as chronic inflammation, alcohol consumption and tobacco smoking can affect on it [15]. In addition, microenvironmental decline, progressive decline of competitive fitness of the tissue, and relatively low levels of clearing altered (dysfunctional) cells have been seen in with aging tissues, some of which can be oncogenic [17]. In the case of the brain during somatic evolution, cellular division/differentiation is relatively slow or almost quiescent, thus mutations that occur early in development are less frequent, but remain high in VAF. On the other hand, in the case of blood and other tissues, continuous differentiation proceeds, and if positive selection does not occur due to a special driver mutation, it can be speculated that it will have a relatively low VAF with a high number of mutations while having similar cellular fitness. (page 15, line 295-308, revised manuscript)

All in all, the present manuscript provides an important contribution to cutting edge research on genetic mosaicism, which is getting a lot of attention now, but extending the present research to incorporate the above questions could help to shed light on the mechanisms underlying the observed mutational profiles.

**Reference**

1.	Bettegowda C, Sausen M, Leary RJ, Kinde I, Wang Y, Agrawal N, et al. Detection of circulating tumor DNA in early- and late-stage human malignancies. Sci Transl Med. 2014;6(224):224ra24. Epub 2014/02/21. doi: 10.1126/scitranslmed.3007094. PubMed PMID: 24553385; PubMed Central PMCID: PMCPMC4017867.

2.	Siravegna G, Mussolin B, Venesio T, Marsoni S, Seoane J, Dive C, et al. How liquid biopsies can change clinical practice in oncology. Ann Oncol. 2019;30(10):1580-90. doi: 10.1093/annonc/mdz227.

3.	Lo YD, Zhang J, Leung TN, Lau TK, Chang AM, Hjelm NM. Rapid clearance of fetal DNA from maternal plasma. The American Journal of Human Genetics. 1999;64(1):218-24.

4.	Merker JD, Oxnard GR, Compton C, Diehn M, Hurley P, Lazar AJ, et al. Circulating Tumor DNA Analysis in Patients With Cancer: American Society of Clinical Oncology and College of American Pathologists Joint Review. J Clin Oncol. 2018;36(16):1631-41. Epub 2018/03/06. doi: 10.1200/jco.2017.76.8671. PubMed PMID: 29504847.

5.	Zill OA, Banks KC, Fairclough SR, Mortimer SA, Vowles JV, Mokhtari R, et al. The Landscape of Actionable Genomic Alterations in Cell-Free Circulating Tumor DNA from 21,807 Advanced Cancer Patients. Clin Cancer Res. 2018;24(15):3528-38. Epub 2018/05/20. doi: 10.1158/1078-0432.Ccr-17-3837. PubMed PMID: 29776953.

6.	de Haan G, Lazare SS. Aging of hematopoietic stem cells. Blood. 2018;131(5):479-87. Epub 2017/11/17. doi: 10.1182/blood-2017-06-746412. PubMed PMID: 29141947.

7.	Natarajan P, Jaiswal S, Kathiresan S. Clonal Hematopoiesis: Somatic Mutations in Blood Cells and Atherosclerosis. Circ Genom Precis Med. 2018;11(7):e001926. Epub 2018/07/11. doi: 10.1161/circgen.118.001926. PubMed PMID: 29987111; PubMed Central PMCID: PMCPMC6082163.

8.	Li R, Di L, Li J, Fan W, Liu Y, Guo W, et al. A body map of somatic mutagenesis in morphologically normal human tissues. Nature. 2021;597(7876):398-403. Epub 2021/08/27. doi: 10.1038/s41586-021-03836-1. PubMed PMID: 34433965.

9.	Alexandrov LB, Kim J, Haradhvala NJ, Huang MN, Tian Ng AW, Wu Y, et al. The repertoire of mutational signatures in human cancer. Nature. 2020;578(7793):94-101. Epub 2020/02/07. doi: 10.1038/s41586-020-1943-3. PubMed PMID: 32025018; PubMed Central PMCID: PMCPMC7054213 patent applications related to MuTect, ABSOLUTE, MutSig, MSMuTect and POLYSOLVER. All the other authors have no competing interests.

10.	Lodato Michael A, Rodin Rachel E, Bohrson Craig L, Coulter Michael E, Barton Alison R, Kwon M, et al. Aging and neurodegeneration are associated with increased mutations in single human neurons. Science. 2018;359(6375):555-9. doi: 10.1126/science.aao4426.

11.	Bae T, Tomasini L, Mariani J, Zhou B, Roychowdhury T, Franjic D, et al. Different mutational rates and mechanisms in human cells at pregastrulation and neurogenesis. Science. 2018;359(6375):550-5. doi: 10.1126/science.aan8690.

12.	Lee-Six H, Øbro NF, Shepherd MS, Grossmann S, Dawson K, Belmonte M, et al. Population dynamics of normal human blood inferred from somatic mutations. Nature. 2018;561(7724):473-8. doi: 10.1038/s41586-018-0497-0.

13.	Brunner SF, Roberts ND, Wylie LA, Moore L, Aitken SJ, Davies SE, et al. Somatic mutations and clonal dynamics in healthy and cirrhotic human liver. Nature. 2019;574(7779):538-42. doi: 10.1038/s41586-019-1670-9.

14.	Walsh C, Choudhury S, Huang A, Kim J, Morillo K, Maury E, et al. Somatic mutations in single human cardiomyocytes demonstrate accelerated age-related DNA damage and cell fusion2020.

15.	Kakiuchi N, Ogawa S. Clonal expansion in non-cancer tissues. Nat Rev Cancer. 2021;21(4):239-56. Epub 2021/02/26. doi: 10.1038/s41568-021-00335-3. PubMed PMID: 33627798.

16.	Swiatczak B. Struggle within: evolution and ecology of somatic cell populations. Cellular and Molecular Life Sciences. 2021;78(21):6797-806. doi: 10.1007/s00018-021-03931-6.

17.	Laconi E, Marongiu F, DeGregori J. Cancer as a disease of old age: changing mutational and microenvironmental landscapes. Br J Cancer. 2020;122(7):943-52. doi: 10.1038/s41416-019-0721-1.
