# Supplementary material for: Analysis of low-level somatic mosaicism reveals stage and tissue-specific mutational features in human development
Source: PLoS Genet. 2022 Sep 19;18(9):e1010404. doi: 10.1371/journal.pgen.1010404 (PMC9560606; doi:10.1371/journal.pgen.1010404)
Supplement: Attachment — Submitted filename: Response to Reviews_0802_KJH.docx [file pgen.1010404.s005.docx]

**-Response to Reviews-**

**Reviewer #1**

**General Comment:** The authors have made a substantial effort to address my questions and concerns in the responses to reviewers and have added a very extensive paragraph about the limitations of the study in the Discussion section, which I appreciate. However, I believe the manuscript still lacks some of the necessary amendments.

**SUMMARY of the response to the reviewer 1:**

We thank the reviewers for their thoughtful comments and point-by-point responses to the Reviewer’s comments are given below. Changes to the manuscript have been highlighted in yellow in the revision.

**Q1.** The first one relates to the problem of different ages for the different sample types, which is obvious from Fig. 4C. The authors provided an analysis restricted to patients aged 50 to 75 in the responses to reviewers. I believe this data should be discussed in the text and included as a supplementary figure.

**Response to Q1:** We thank the reviewer for the valuable comments. As your comment, tissues included in our study showed uneven distribution by age. In the case of brain, diverse age groups were included, whereas in the case of blood, the samples were from patients younger than 20 years old and over 50 years old, and for other organs, samples younger than 50 years old were included. Since we had age-matched patients aged 50-75 years, we compared the numbers and VAF of mutations in this age group. Despite the sampling issues, the molecular characteristics of late-stage mutations were maintained in the 50- to 75-year-old group, which was directly comparable. Therefore, we revised the manuscript and supplementary figure to clarify the issue.

1. Another issue includes the uneven distribution of organs by age. For the brain, various age groups were included, whereas, for blood samples, there were many samples from patients who were under 20 and over 50 years old. For other organs, samples from patients in age groups under 50 years were included. Therefore, the number and VAFs for late-somatic mutations were not compared using age-matched tissue groups. We extracted data from patients aged 50–75 years, which was directly comparable in all three groups, and the molecular characteristics of late-stage mutations were maintained (e.g., fewer mutations and high VAF in the brain group, moderate mutations and VAF in other organ groups, and high mutation rates with low VAF in the blood group (Supplementary figures 1E and 1F)). Despite sampling limitations, our data was representative of late-stage somatic mutations. (Page 15, line 300−Page 16, line 310, revised manuscript)
2. **Supplementary Fig 1** Late mutational analysis by mutagen in different organs (brain, blood, other organs) (A), (B) Age correlation with somatic mutation VAFs in late-stage variants and blood late-stage variants. (C) VAFs of late-stage somatic mutations in brains are classified by the diseases in individuals. (D) VAFs of late-stage somatic mutations in the heart, liver, and lipoid tissues. (E), (F) Number of mutations per individual and VAF distribution in individuals aged 50–75 years. (Supplementary Fig 1 legend, revised manuscript)

**Q2.** The second issue relates to the selection of tissues for the study, which was done based on convenience. This leads to the “other organs” being a random mixture of tissues, which is still not well explained in the article, and to the brain tissue including a large proportion of samples from diseased tissue. The article would greatly benefit from more clarity on the types of samples included and from additional plots (which could be included as supplementary data) in which the authors further analyze the data to demonstrate that the choice of samples is not biasing the results. For example, in response to Reviewer #2, the authors indicate that the VAF of disease tissues is not different from the normal. All those comparisons should be clearly presented with dot plots in the article. Also, the authors have included this sentence in results: “VAF of late-stage mutations was inversely correlated with age, probably due to the accumulation of late-somatic mutations in blood with a low allelic fraction with aging”. This plot should be shown for all late-stage mutations and also for blood mutations only.

**Response to Q2:** We thank the reviewer for the valuable comments. We discussed that in depth with our co-authors. In recent studies, it has been well known that the frequency and VAF of somatic/mosaic mutation vary by tissue [1-3]. Therefore, it is difficult to have a representative set of normal tissues of other organs, and in order to truly remove the bias, it is necessary to collect a large number of types of tissue from all organs. However, this is not practically possible thus the issues with the normal tissue selection is inevitable. Therefore, we clearly explain the composition of the “other organ” in our study, and clarify that the results of the analysis were obtained with respect to the selection and composition of the organ as below.

1. An important consideration includes those somatic mutations should vary not only in cell types and tissues, but also in developmental stages, even in healthy individuals [39-41]. Therefore, it is difficult to have a representative set of normal tissues of other organs. To eliminate this bias, it is necessary to collect several types of tissues from all organs. Our study had a selection bias because age-mismatched “other organs” were a mixture of different organs, whereas brain tissues contained various locations with diverse ages, but diseased brain tissues were obtained from some patients. This can lead to erroneous results, and therefore, molecular properties were compared by subdividing tissues based on other organ compositions and types of brain disease (Supplementary Fig 1C and 1D). Although there were differences in VAFs for each brain disease, all organs showed high VAFs compared to other organs or blood groups, as described above (Supplementary Fig 1C). In other organ groups, the VAFs of the heart, liver, and lipoid tissues were 2.88, 1.56, and 0.96, respectively, and there was a statistically significant difference between heart and liver samples (*p*-value = 0.03715, Wilcoxon rank sum test) (Supplementary Fig 1D). Nevertheless, the difference in mean VAF values was within 1.32%, and the composition of other organs in this study had little effect on the conclusions. (Page 15, line 285-300, revised manuscript)
2. **Supplementary Fig 1** Late mutational analysis by mutagen in different organs (brain, blood, other organs) (A), (B) Age correlation with somatic mutation VAFs in late-stage variants and blood late-stage variants. (C) VAFs of late-stage somatic mutations in brains are classified by the diseases in individuals. (D) VAFs of late-stage somatic mutations in the heart, liver, and lipoid tissues. (E), (F) Number of mutations per individual and VAF distribution in individuals aged 50–75 years. (Supplementary Fig 1 legend, revised manuscript)

**Q3.** Finally, the article still requires attention to the explanation of the figures and methods. **(1)** What are the numbers in the bars of Fig. 1A? what do red and blue colors indicate? **(2)** In Fig. 3B, how are the relative contributions calculated? **(3)** In Methods, there are some sentences that are hard to understand. For example: “In the filtering process of paired-mode somatic calling using Mutect2, “normal artifact” is corresponding to it; variants are non-germline mutation, differ from reference sequence, and contained in matched pair samples. **(4)** “Overall, the article would benefit from careful reading to improve some language issues and to make some of the explanations in methods and results clearer.

**Response to Q3:** We thank the reviewer for the valuable comments. **(1)** We described schematic figure of shared and organ-specific mutations. In lower right panel in Fig 1A, the colored bar means total reads (reference and altered reads). Red- and blue- bar described reference and altered reads, respectively. We thought that our description might cause confusion to readers, therefore we revised it in the revised manuscript.

**(1)** After strict filtration and tests for organ specificity using one-sample proportion tests, the shared and organ-specific mutations were classified. The colored bar indicates read distribution, and the red and blue-colored bars indicate the reference and altered reads, respectively (lower right panel). (Figure 1 legend, revised manuscript)

**(2)** Mutations from three groups were extracted with three signatures by novel signature extraction. Contributions were obtained by fitting the extracted signatures to the mutations of each group. These analyzes used mutational patterns (R package). We added this comment in Fig 3B legend.

1. Non-negative matrix factorization-based novel signature extraction (NMF, 200 iterations) and transcriptional strand-bias analysis were performed using the Mutational Patterns program (R package). (Page 23, line 453-455, revised manuscript)
2. Mutations from the three groups were extracted as three signatures by NMF. Mutational contributions were obtained by fitting the extracted signatures to the mutations in each group. (Figure 3 legend, revised manuscript)

**(3)** To better understanding, we deleted that comments in the revised manuscript.

1. In the filtering process, paired-mode somatic calling by Mutect2 was used. (Page 22, line 429-430, revised manuscript)

**(4)** We will improve the language issue through language editing service before re-submission. Thank you so much for the comment

**Reviewer #2**

**Q1.** Hyeonju Son and associates have revised and substantially improved their analysis of somatic mosaicism using deep exome sequencing. They have adequately addressed all of my previous major concerns. There are still numerous errors of English grammar and word usage; this paper would benefit greatly from copy editing for English usage.

**Response to Reviewer #2 :** We thank the reviewer for the valuable comments. We improve the quality of our manuscript through language editing service before re-submission. Thank you so much for your suggestion.

**Reviewer #3**

**Response to Reviewer #3 :** We thank the reviewer very much for the kind comments.

**Reference**

1.	Li R, Di L, Li J, Fan W, Liu Y, Guo W, et al. A body map of somatic mutagenesis in morphologically normal human tissues. Nature. 2021;597(7876):398-403. Epub 2021/08/27. doi: 10.1038/s41586-021-03836-1. PubMed PMID: 34433965.

2.	García-Nieto PE, Morrison AJ, Fraser HB. The somatic mutation landscape of the human body. Genome Biol. 2019;20(1):298. doi: 10.1186/s13059-019-1919-5.

3.	Kakiuchi N, Ogawa S. Clonal expansion in non-cancer tissues. Nat Rev Cancer. 2021;21(4):239-56. Epub 2021/02/26. doi: 10.1038/s41568-021-00335-3. PubMed PMID: 33627798.
